# IGF-1 receptor and IGF binding protein-3 might predict prognosis of patients with resectable pancreatic cancer

**DOI:** 10.1186/1471-2407-13-392

**Published:** 2013-08-21

**Authors:** Toshiki Hirakawa, Masakazu Yashiro, Akihiro Murata, Keiichiro Hirata, Kenjiro Kimura, Ryosuke Amano, Nobuya Yamada, Bunzo Nakata, Kosei Hirakawa

**Affiliations:** 1 Department of Surgical Oncology, Osaka City University Graduate School of Medicine, 1-4-3 Asahi-machi, Osaka, Abeno-ku, Japan; 2 Oncology Institute of Geriatrics and Medical Science, Osaka City University Graduate School of Medicine, 1-4-3 Asahi-machi, Osaka, Abeno-ku 545-8585, Japan

**Keywords:** Pancreatic cancer, IGF1R, IGFBP3, Prognosis

## Abstract

**Background:**

The present study aimed to elucidate the clinicopathologic role of insulin-like growth factor-1 receptor (IGF1R) and IGF binding protein-3 (IGFBP3) in patients with pancreatic cancer. The function of IGFBP3 is controversial, because both inhibition and facilitation of the action of IGF as well as IGF-independent effects have been reported. In this study, IGF1R and IGFBP3 expression was examined, and their potential roles as prognostic markers in patients with pancreatic cancer were evaluated.

**Methods:**

Clinicopathological features of 122 patients with curatively resected pancreatic cancer were retrospectively reviewed, and expression of IGF1R and IGFBP3 was immunohistochemically analyzed.

**Results:**

Expression of IGF1R and IGFBP3 was observed in 50 (41.0%) and 37 (30.3%) patients, respectively. IGF1R expression was significantly associated with histological grade (*p* = 0.037). IGFBP3 expression had a significant association with tumor location (*p* = 0.023), and a significant inverse association with venous invasion (*p* = 0.037). Tumors with IGF1R-positive and IGFBP3-negative expression (n = 32) were significantly frequently Stage II and III (*p* = 0.011). The prognosis for IGF1R positive patients was significantly poorer than that for IGF1R negative patients (*p* = 0.0181). IGFBP3 protein expression did not correlate significantly with patient survival. The subset of patients with both positive IGF1R and negative IGFBP3 had worse overall survival (8.8 months versus 12.6 months, respectively, *p* < 0.001).

**Conclusion:**

IGF1R signaling might be associated with tumor aggressiveness, and IGFBP3 might show antiproliferative effects in pancreatic cancer. Both high IGF1R expression and low IGFBP3 expression represent useful prognostic markers for patients with curatively resected pancreatic cancer.

## Background

Pancreatic ductal adenocarcinoma (PDAC) is one of the most lethal solid tumors, and carries an extremely poor prognosis [1]. Although the management and treatment of patients with pancreatic cancer have improved over the last few decades, the overall 5-year survival rate remains less than 5% [2]. Long-term survival is rare, even in patients who undergo a histologically curative operation, with overall 5-year survival rates ranging from 10% to 25% [3,4]. The high mortality rate associated with pancreatic cancer is known to be due to extensive invasion into surrounding tissues and metastasis to distant organs at the time of diagnosis (or even after a curative operation); however, the molecular mechanisms of the highly aggressive nature of PDAC remains unclear [5].

Previous studies have shown an association between progression of PDAC and overexpression of several growth factors (and their receptors) including insulin-like growth factor (IGF), vascular endothelial growth factor (VEGF), fibroblast growth factor (FGF), and platelet-derived growth factor (PDGF) [6-8]. Most of the cellular effects of IGF-I and IGF-II are mediated by the IGF-I receptor (IGF1R). Binding of the ligand to IGF1R leads to tyrosine phosphorylation of the major receptor substrate followed by activation of certain downstream signaling cascades [9]. The IGFs have been implicated through IGF1R in the pathogenesis, cell proliferation, and cell survival of many cancers [10,11]. IGF-1, which is produced primarily by the liver, is known to play an important role in the regulation of cell proliferation, differentiation, and apoptosis [10-12]. Clinical studies in colorectal, esophageal, and pancreatic cancers have shown that IGF1R signaling correlates with increased tumor growth and malignancy in vitro [8,13,14].

The IGF system is a complex network of growth factors (IGF-I and IGF-II), cell surface trans-membrane receptors (IGF1R), and high affinity IGF-binding proteins (IGFBPs) that play an important role in normal cellular growth and development, and disruption of the balance of this system has been implicated in the etiology and progression of breast and other cancers [15]. Activation of the IGF system stimulates proliferation, differentiation, angiogenesis, metastasis, survival, and resistance to anticancer therapies in many cancers [16], supporting the idea that the IGF system is an attractive therapeutic target. The biological actions of IGFs are modulated by a family of IGFBPs in the local tissue microenvironment [17,18]. IGFBP3 is part of the family of six IGFBPs that bind the peptide growth factors IGF-I and IGF-II with high affinity and regulate their bioactivity [19]. IGFBP3 is the most abundant IGFBP, being present in almost all tissues. IGFBP3 inhibits IGF1R mediated signaling by preventing the interaction of IGFs with IGF1R. IGFBP3 regulates the mitogenic action of IGFs or inhibits their antiapoptotic effects through IGF-dependent and IGF-independent mechanisms [20,21]. However, there are few evidences of an association between IGFBP3 and enhanced cell proliferation. These findings have encouraged investigators to investigate whether IGFBP3 plays a positive or negative role in IGF-promoted tumor development.

Although serum levels of IGF-I are generally considered to be a positive risk factor for development of colorectal cancer, the role of IGFBP3 appears less clear. Both the inhibition and activation of cellular functions by these proteins have been demonstrated to depend on cell type [22]. The present study examined IGF1R and cell surface-associated IGFBP3 expression in patients with pancreatic cancer.

## Methods

### Patients

A total of 122 patients who had undergone resection of a primary pancreatic tumor at the Department of Surgical Oncology, Osaka City University Hospital were included. The pathologic diagnoses and classifications were made according to the UICC Classification of Malignant Tumors [23]. No patients had hematogenous metastases or peritoneal dissemination before surgery. Histological findings are according to the classification of pancreatic carcinoma in Japan Pancreas Society [24]. Patients’ characteristics are shown in Table 1. The median age of patients was 68 years (range 33–84 years). A total of 79 patients (64.8%) died during the follow-up period, and the majority of patients were male (67.2%), and Stage II (78.7%). The observation period is overall survival time that was set in days as the period from the time of resection until the time of death. The study protocol conformed to the ethical guidelines of the Declaration of Helsinki (1975). This study was approved by the Osaka City University ethics committee. Informed consent was obtained from all patients prior to entry.

**Table 1:** Patients’ clinicopathological characteristics

**Clinicopathologic characteristics**	**n = 122**
Gender	
Male	66
Female	56
Age (years)	
Median	68
Range	33-84
Tumor location	
proximal	81
distal	41
Tumor differentiation	
Grade 1	35
Grade 2	67
Grade 3	17
Grade 4	3
Tumor stromal volume	
Medullary type (med)	1
Intermediate type (int)	85
Scirrhous type (sci)	36
T category	
T1	11
T2	15
T3	84
T4	12
N category	
N0	56
N1	66
Stage	
I	15
II	95
III	12
IV	0

TNM classification is according to the International Union against Cancer (UICC, 2003).

Medullary type (med): scanty stroma, Intermediate type (int): the quantity of stroma is intermediate between the two above types, Scirrhous type (sci): abundant stroma.

### Immunohistochemical techniques

Sections of paraffin-embedded tissue (4-μm thick) were prepared. Immunohistochemical staining for IGF1R and IGFBP3 was performed using the avidin-biotin-peroxidase complex method. In brief, the deparaffinized and hydrated tissues were heated for 10 min at 105°C in Target Retrieval Solution (Dako, Carpinteria, CA, USA). Then, the slides were allowed to cool for 20 min on a lab bench in the Target Retrieval Solution at 25°C. The slides were incubated overnight at 4°C with 5 μg/mL of antihuman IGF1R mouse monoclonal antibody (Abcam, Cambridge, MA, USA) and 5 μg/mL of antihuman IGFBP3 rabbit polyclonal antibody (Abcam, Cambridge, MA, USA).

### Immunohistochemical determination

All slides were examined by two of the authors who were blinded to clinical data. The final evaluation of ambiguous cases was decided after discussion between the two authors. For determination of IGF1Rand IGFBP3 protein immunoreactivity, the cytoplasm and membrane staining intensity and patterns were evaluated according to the following scale. Immunoreactivity for IGF1R was evaluated in the neoplastic epithelial cells using a combined scoring system based on the sum of the staining intensity and the percentage of positive cells. Scores from 0–3 were given for the staining intensity and the percentage of positive cells as follows: score of 0, no staining is observed, or is observed in less than 10% of the tumor cells; score of 1+, weak staining is detected in 10% or more of the tumor cells; score of 2+, moderate staining is observed in 10% or more of the tumor cells; and score of 3+, strong staining is observed in 10% or more of the tumor cells. Scores of 0 and 1+ were considered to be negative for IGF1R overexpression, while scores of 2+ and 3+ were considered to be positive for IGF1R overexpression. Immunoreactivity for IGFBP3 was evaluated in the neoplastic epithelial cells using a combined scoring system based on the sum of the staining intensity and the percentage of positive cells. For determination of IGFBP3 protein immunoreactivity, staining of antibody was considered positive if >10% of tumor cells were stained.

### Statistical analysis

The χ^2^-test or Fisher’s exact test was used to determine the significance of the differences between the covariates. Survival durations were calculated using the Kaplan-Meier method and were analyzed by the log-rank test to compare the cumulative survival durations in the patient groups. The Cox proportional hazards model was used for the univariate and multivariate analyses. All analyses were performed using SPSS software (SPSS Japan, Tokyo, Japan). A *P*-value < 0.05 was considered to represent statistical significance.

## Results

### Expression of IGF1R and IGFBP3

**Figure 1:**
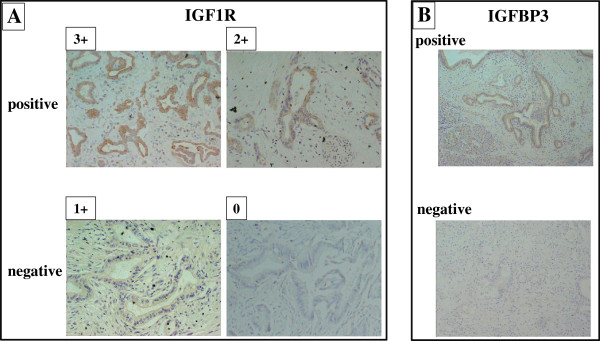
**IGF1R and IGFBP3 expression in pancreatic cancer. A**, Representative IGF1R staining quantified with scores of 0 to 3+ according to staining intensity. (Original magnification X 200). IGF1R was mainly expressed in the cytoplasm of pancreatic cancer cells. **B**, IGFBP3 was expressed in the cell membrane and the cytoplasm of pancreatic cancer cells.

Tumors with positive IGF1R protein showed cytoplasmic staining. Typical images of positive immunostaining for IGF1R in cancer cells are shown in Figure 1A. Overall, seven cases had a score of 0, 69 cases had a score of 1+, 23 cases had a score of 2+, and 27 cases had a score of 3+. Thus, 50 cases (41%) were positive for IGF1R overexpression. Most of the positive staining was observed in the cytoplasm, while two cases showed positive staining in both membranes and cytoplasm. In contrast, no or weak staining was seen in the cytoplasm of pancreatic duct cells and acinar cells, and there was no staining in the membranes. Figure 1B shows a representative picture of IGFBP3 staining. IGFBP3 was mainly expressed in the cytoplasm of cancer cells. Eighty-five cases of PDAC showed negative IGFBP3 expression, whereas 37 cases were positive.

### Clinicopathological association of IGF1R and IGFBP3 expression

Table 2 shows the association of clinicopathological characteristics and IGF1R or/and IGFBP3 expression. IGF1R expression had a significant association with histological grade (Fisher’s exact test, *p* = 0.037). Stromal volume tended to be more abundant in PDAC with IGF1R overexpression, but no significant difference was observed (χ^2^ test, *p* = 0.087). IGFBP3 expression had a significant association with tumor location (χ^2^ test, *p* = 0.023), and a significant inverse association with venous invasion (Fisher’s exact test, *p* = 0.037). IGFBP3 expression tended to be frequent in differentiated PDAC in histological grade, but no significant difference was observed (χ^2^ test, *p* = 0.082).

**Table 2:** **Association between IGF1R** &**IFGBP3 expression and clinicopathological factors in resectable pancreatic cancer**

	**IGF1R expression**			**IGFBP3 expression**			**Both IGF1R (+) and IGFBP3 (−)**		
**Characteristics**	**positive**	**negative**		**positive**	**negative**		**positive**	**others**	
	**n = 50**	**n = 72**	***p-*value**	**n = 37**	**n = 85**	***p-*value**	**n = 32**	**n = 90**	***p-*value**
Age									
<60	7	18		10	15		3	22	
≧60	43	54	0.139	27	70	0.238	29	68	0.07
Gender									
Male	25	31		15	41		16	40	
Female	25	41	0.449	22	44	0.433	16	50	0.588
T category									
T1, T2	9	17		7	19		3	23	
T3, T4	41	55	0.457	30	66	0.670	29	67	
N category									0.077
N0	23	33		20	36		14	42	
N1	27	39	0.986	17	49	0.233	18	48	0.776
Stage									
I	4	11		5	10		0	15	
II & III	46	61		32	75		32	75	
Tumor location			0.273			0.787			0.011
proximal	31	50		30	51		18	63	
distal	19	22		7	34		14	27	
Tumor differentiation			0.392			0.023			0.157
Grade1, 2	46	56		34	68		28	74	
Grade 3, 4	4	16	0.037	3	17	0.082	4	16	0.588
Lymphatic invasion									
negative	24	42		19	47		15	51	
positive	26	30	0.260	18	38	0.688	17	39	0.340
Arterial invasion									
negative	43	67		33	77		27	83	
positive	7	5	0.198	4	8	0.754	5	7	0.200
Venous invasion									
negative	45	62		36	71		28	79	
positive	5	10	0.52	1	14	0.037	4	11	0.967
Intrapancreatic nerve invasion									
negative	21	38		16	43		16	43	
positive	29	34	0.241	21	42	0.456	16	47	0.829
Tumor stromal volume									
med & int	31	55		27	59		19	67	
sci	19	17	0.087	10	26	0.692	13	23	0.108
IGFBP3 expression									
negative	32	53							
positive	18	19	0.256						

Medullary type (med): scanty stroma, Intermediate type (int): the quantity of stroma is intermediate between the two above types, Scirrhous type (sci): abundant stroma.

### Relationship between clinicopathological features and tumors with IGF1R-positive and IGFBP3-negative expression

Among the 50 patients with positive IGF1R expression, 32 patients (64.0%) had negative IGFBP3 expression. Tumors with IGF1R-positive and IGFBP3-negative expression (n = 32) were significantly frequently found to have Stage II and III cancer (χ^2^ test, *p* = 0.011) compared to the other groups (n = 90). Tumors with IGF1R-positive and IGFBP3-negative expression tended to be in older patients (Fisher’s exact test, *p* = 0.07) and advanced T stage (χ^2^ test, 0.077). Among the 72 patients with negative IGF1R, 53 patients (73.6%) showed negative IGFBP3 expression, whereas 19 patients (26.4%) had positive IGFBP3 expression. No association was found between IGF1R and IGFBP3 expression.

### Survival

**Figure 2:**
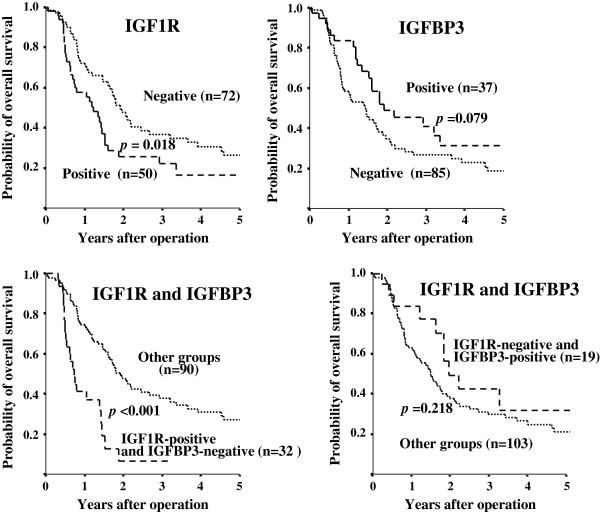
**Overall survival of patients based on IGF1R and IGFBP3 expression.** The survival curve shows the Kaplan-Meier overall survival curves in relation to the IGF1R and IGFBP3 levels in patients with pancreatic cancer. A statistically significant difference in survival was observed between patients with IGF1R-positive and IGF1R-negative tumors (p = 0.018). The prognosis of patients with IGF1R-positive and IGFBP3-negative patients showed a significant correlation with overall survival (p < 0.001). IGFBP3 expression alone tended to be associated with overall survival (p = 0.079). The co-expression of IGF1R-negative and IGFBP3-positive PDAC was not associated with overall survival (*p* = 0.218).

**Figure 3:**
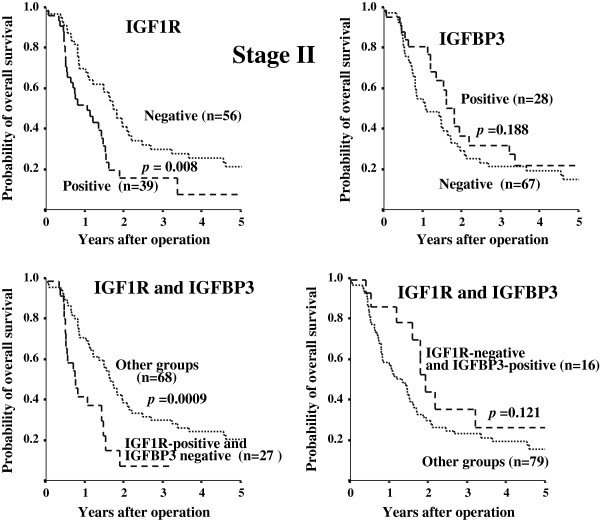
**Overall survival stratified by IGF1R and IGFBP3 expression in cancer cells in patients with clinical stage II tumors.** Prognosis of IGF1R-positive cancer was significantly poorer (p = 0.008) than that of IGF1R-negative cancer in the stage II group. Analysis of prognosis of patients with IGF1R-positive and IGFBP3-negative tumors shows a significant correlation with overall survival (p = 0.0009) in patients with stage II tumors.

Kaplan-Meier survival analyses showed a significantly poorer overall survival in the IGF1R-positive group compared to the IGF1R-negative group (p = 0.018). Moreover, the prognosis of patients with IGF1R-positive and IGFBP3-negative PDAC was significantly poorer than that of other patients (p < 0.001). In contrast, the prognosis of patients with IGF1R-negative and IGFBP3-positive PDAC was not significantly correlation with overall survival (*p* = 0.218), while IGFBP3 expression alone tended to be associated with overall survival (p = 0.079) (Figure 2). Figure 3 shows the overall survival stratified for IGF1R and IGFBP3 expression in cancer cells according to clinical stage II status. The prognosis for IGF1R positive patients with stage II tumors was significantly (*p* = 0.0080) poorer than that for IGF1R negative patients, while no significant difference in the prognosis was found between the IGF1R expression in either stage I or III tumors (data not shown). On univariate analysis, three factors, IGF1R overexpression, IGF1R-positive and IGFBP3-negative expression, and lymph node metastasis, were significantly associated with worse overall survival. Because IGF1R status is deeply associated with IGF1R and IGFBP3 status, multivariate analysis was performed with two factors: IGF1R-positive and IGFBP3-negative expression, and lymph node metastasis. The multivariate survival analysis indicated that IGF1R-positive and IGFBP3-negative expression, along with lymph node metastasis, were independent prognostic indicators (Table 3). IGF1R-positive and IGFBP3-negative expression and lymph node metastasis were independent predictors of poor prognosis.

**Table 3:** Univariate and multivariate survival analyses in pancreatic cancer

**Variable**	**Univariate analysis**			**Multivariate analysis**		
	**Hazard ratio**	**95% CI**	***p*-value**	**Hazard ratio**	**95% CI**	***p*-value**
IGF1R expression						
Positive vs negative	1.714	1.091-2.693	0.020			
IGFBP3 low expression						
Positive vs negative	1.561	0.946-2.576	0.081			
IGF1R (+) & IGFBP3 (−)						
Positive vs negative	3.101	1.843-5.218	< 0.001	3.060	1.823-5.138	< 0.001
Gender						
male vs female	0.975	0.623-1.525	0.911			
Age						
≧ 60 vs <60	1.444	0.822-2.537	0.201			
T category						
3, 4 vs 1,2	1.844	0.997-3.413	0.051			
Lymph node metastasis						
Positive vs negative	1.79	1.106-2.736	0.017	1.718	1.092-2.702	0.019
Tumor location						
proximal vs distal	1.108	0.671-1.736	0.752			
Lymphatic invasion						
Positive vs negative	1.332	0.855-2.075	0.205			
Arterial invasion						
Positive vs negative	1.209	0.554-2.637	0.634			
Venous invasion						
Positive vs negative	1.579	0.812-3.071	0.178			
Intrapancreatic nerve invasion						
Positive vs negative	1.452	0.930-2.267	0.101			
Tumor differentiation						
grade 1,2 vs grade 3,4	1.452	0.826-2.551	0.195			
Tumor stromal volume						
med/int vs scir	0.724	0.453-1.156	0.176			

## Discussion

The present study analyzed the immunohistochemical expression of IGF1R and IGFBP3 with clinicopathological variables and the correlation with overall survival in 122 patients with PDAC. IGF1R expression had a significant association with histological grade of tumor differentiation, and also tended to be associated with abundant stroma. These findings suggest that the IGF1R signaling system might be correlated with histopathologic features of PDAC. It has been reported that IGF1 is produced from stromal cells [11]. There might be an interaction between cancer cells and stromal cells via IGF/IGF1R signaling. PDAC patients with IGF1R-positive expression showed significantly poorer survival, compared to the IGF1R-negative group (Figure 2). The present findings suggest that the IGF1R signaling system might be correlated with tumor aggressiveness in PDAC, as has been previously reported [25,26].

IGF bioavailability is regulated by a family of six IGF-binding proteins (IGFBP), of which IGFBP3 is the major IGF carrier protein [17]. The function of IGFBP3 is controversial. IGFBP3 has been shown to produce either inhibition [27-29] or potentiation [30-32] of IGF effects. The direction of the effect may depend on the cell type [27]. In this study, favorable survival in the IGFBP3-positive group was noted, but statistical significance was not obtained (Figure 2). IGFBP3 expression had an inverse association with venous invasion. These findings suggest that IGFBP3 might show antiproliferative effects in PDAC. IGFBP3 expression had a significant association with proximal tumors. Most insulin is secreted from the distal pancreas. IGFBP3 expression might be associated with lesions involving insulin secretion.

Next, the significance of the combination of IGF1R expression and IGFBP3 expression was evaluated. Tumors with IGF1R-positive and IGFBP3-negative expression were significantly frequently found at an advanced clinical stage (II or III), compared to the other groups. The prognosis of patients with IGF1R-positive and IGFBP3-negative PDAC was poorer than that of other groups, especially in patients with stage II tumors (Figure 3). The IGF1R-positive and IGFBP3-negative subgroup was the group with the worst prognosis (Figures 2 &3). These findings suggest that IGFBP3 could produce inhibition of IGF effects. Decreased IGFBP3 production and increased IGF1R expression in pancreas tumors might enhance the tumorigenesis and cell motility as previously reported [26,33,34]. Prediction of prognosis in patients with operable PDAC is important to determine the adjuvant therapy. This is especially true in patients with stage II tumors, because the local recurrence rate of PDAC is high, even in patients with curative R0 operations. The present study suggests that combined evaluation of IGF1R expression and IGFBP3 expression is a useful prognostic factor in pancreatic cancer, especially with clinical stage II tumors.

Although IGFBP3 is the major IGF carrier protein, some paper reported that IGFBP3 has IGF-independent antiproliferative and proapoptotic effects [20,21]. The inhibition of IGF1-induced functions by cell surface-associated IGFBP3 have been reported [27,29]; however, the relationship between membrane-associated IGFBPs and IGF1R signaling is less well understood. Therefor significance of co-expression of IGFBP3 and IGF1R in PDAC remains obscure. We then analyzed the significance of IGF1R-negative and IGFBP3-positive group with respect to overall survival (in the right bottom diagram of Figures 2 &3), which might clarify whether IGFBP3 is IGF1/IGF1R signaling-independent or not. Although IGFBP3 expression alone tended to be associated with overall survival (p = 0.079), co-expression of IGF1R-negative and IGFBP3-positive PDAC was not associated with overall survival (*p* = 0.218). These data suggested that the function of IGFBP3 might be dependent on IGF1R expression.

## Conclusion

IGF1R signaling might be associated with tumor aggressiveness, and IGFBP3 might show antiproliferative effects in pancreatic cancer. Both high IGF1R expression and low IGFBP3 expression represent useful prognostic markers for patients with curatively resected pancreatic cancer.

## Competing interests

All of the authors have no conflicts of interest to disclose.

## Authors’ contributions

TH: study design, data analysis, material sampling, paper preparation. MY: study design, data analysis, interpretation of data, paper preparation. AM, KH, KK, RA, NY and BN: material sampling. KH: data analysis, interpretation. All authors read and approved the final manuscript.

## Pre-publication history

The pre-publication history for this paper can be accessed here:

http://www.biomedcentral.com/1471-2407/13/392/prepub
